# Hemodynamics-driven mathematical model of first and second heart sound generation

**DOI:** 10.1371/journal.pcbi.1009361

**Published:** 2021-09-22

**Authors:** Mehrdad Shahmohammadi, Hongxing Luo, Philip Westphal, Richard N. Cornelussen, Frits W. Prinzen, Tammo Delhaas

**Affiliations:** 1 Department of Biomedical Engineering, Cardiovascular Research Institute Maastricht (CARIM), Maastricht University, Maastricht, The Netherlands; 2 Department of Physiology, Cardiovascular Research Institute Maastricht (CARIM), Maastricht University, Maastricht, The Netherlands; 3 Bakken Research Centre, Medtronic, BV, Maastricht, The Netherlands; Stanford University, UNITED STATES

## Abstract

We propose a novel, two-degree of freedom mathematical model of mechanical vibrations of the heart that generates heart sounds in CircAdapt, a complete real-time model of the cardiovascular system. Heart sounds during rest, exercise, biventricular (BiVHF), left ventricular (LVHF) and right ventricular heart failure (RVHF) were simulated to examine model functionality in various conditions. Simulated and experimental heart sound components showed both qualitative and quantitative agreements in terms of heart sound morphology, frequency, and timing. Rate of left ventricular pressure (LV dp/dt_max_) and first heart sound (S1) amplitude were proportional with exercise level. The relation of the second heart sound (S2) amplitude with exercise level was less significant. BiVHF resulted in amplitude reduction of S1. LVHF resulted in reverse splitting of S2 and an amplitude reduction of only the left-sided heart sound components, whereas RVHF resulted in a prolonged splitting of S2 and only a mild amplitude reduction of the right-sided heart sound components. In conclusion, our hemodynamics-driven mathematical model provides fast and realistic simulations of heart sounds under various conditions and may be helpful to find new indicators for diagnosis and prognosis of cardiac diseases.

**New & noteworthy:**

To the best of our knowledge, this is the first hemodynamic-based heart sound generation model embedded in a complete real-time computational model of the cardiovascular system. Simulated heart sounds are similar to experimental and clinical measurements, both quantitatively and qualitatively. Our model can be used to investigate the relationships between heart sound acoustic features and hemodynamic factors/anatomical parameters.

## Author summary

Among various vital signals used for diagnosis and prognosis of cardiac diseases, heart sounds are not employed precisely because physicians subjectively assess their auscultatory findings. On the other hand, recorded heart sounds are also difficult to quantitatively relate to different cardiac conditions given the complex nature of their generation. We therefore employed cardiovascular modeling and developed a novel hemodynamics-driven mathematical model for heart sound generation to unravel the relationships between heart sounds and other vital signals. Simulated and experimental heart sound components showed qualitative and quantitative agreements in terms of heart sound morphology, frequency, and timing, not only during normal conditions, but also during simulated exercise and heart failure. Our model can be used to understand generation of heart sounds in more details and may be helpful to find new diagnostic indicators and treatment methods of cardiac disorders.

## Introduction

Previous studies have proposed various theories about the genesis of heart sounds including sudden tension in otherwise loose valve membranes [1], sudden tension in chordae tendineae [2] and forceful striking of the valve leaflets [3]. A very influential premise about the origin of the major heart sound components was proposed by Rushmer in 1955 [4] who suggested that abrupt cessation of backward flow by each valve closure causes a vibration of the column of blood and the structures surrounding it including valve leaflets as well as the ventricular, atrial and arterial walls. Though this hypothesis on heart sound generation is still well-accepted, up till to date it has been relatively overlooked in modeling studies regarding how hemodynamic factors may contribute to heart sound genesis. Also, a complete real-time model of cardiovascular system including heart sounds generation does not exist.

In the present study, we propose a novel mechanical vibration model of heart sound generation based on Rushmer’s theory [4] and implemented in CircAdapt, a lumped-parameter real-time computational model of cardiovascular system [5]. Simulated hemodynamics in CircAdapt were used to generate heart sounds. A two-degree-of-freedom mathematical model was used to describe the mechanical vibrations of cardiohemic structures. Blood masses trapped on either side of the valves, connected to surrounding viscoelastic materials, were considered as vibrating structures. We used our model to investigate the relation between hemodynamic factors and acoustic features of heart sounds in rest, during exercise and in heart failure.

## Materials and methods

### Ethics statement

The research protocol for the animal experiments were approved by the Central Committee for Animal experiments (CCD) in The Netherlands and the Animal Experimental Committee of Maastricht University.

### Heart sound model

**Fig 1:**
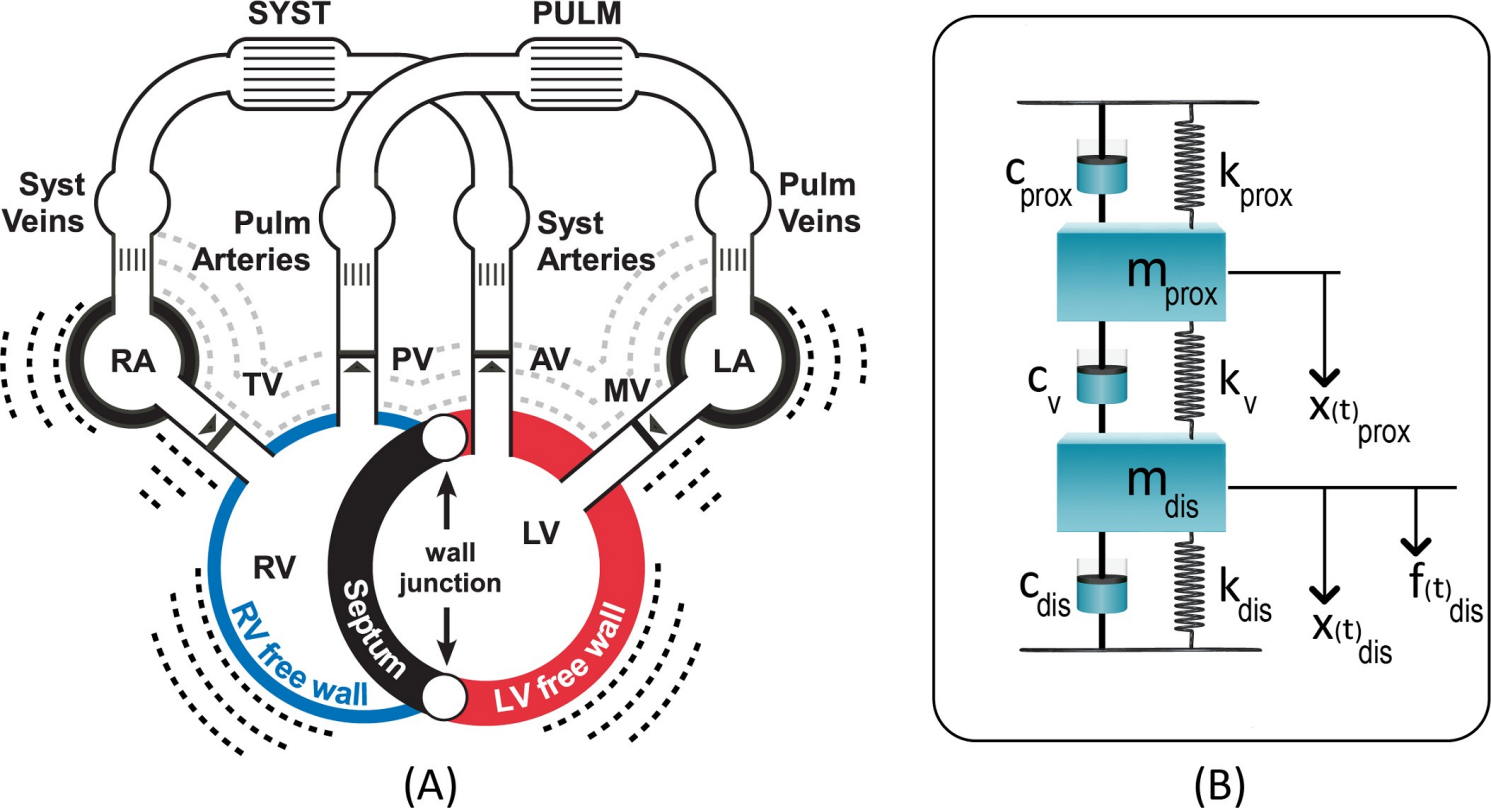
(a) CircAdapt model for simulation of cardiovascular mechanics and hemodynamics. (b) Two-degree- of-freedom model used for heart sound generation and embedded in CircAdapt. For each valve the same model was used albeit with different proximal and distal mechanical and hemodynamic values. Panel (a): **LA**: left atrium, **LV**: left ventricle, **MV**: mitral valve, **PV**: pulmonary valve, **RA**: right atrium, **RV**: right ventricle, **TV**: tricuspid valve. Panel (b): **c**: damping factor, **dis**: distal to valve, **f**: driving force, **k**: spring factor, **m**: blood mass, **prox**: proximal to valve, **v**: valve, **x**: movement of mass.

A module to model the first and second heart sounds by hemodynamic factors was developed in CircAdapt [5], a lumped-parameter, closed-loop model of the human heart and circulation which simulates real-time beat-to-beat mechanics and hemodynamics of the cardiac chambers and blood vessels in both healthy and pathological conditions (**Fig 1A**). According to basic physiological and physical properties, closure of atrio-ventricular and ventriculo-arterial valves is induced by retrograde blood flow and a reversal of the pressure gradients. Abrupt cessation of retrograde blood flow at the moment of actual valve closure causes a vibration in the column of blood and the surrounding viscoelastic structures, (i.e. the valve leaflets and ventricular, atrial and arterial walls), giving rise to the heart sounds. The first heart sound (S1) comprises of a mitral (M1) and tricuspid (T1) valve component whereas the second heart sound (S2) comprises of an aortic (A2) and pulmonary (P2) valve component. A two degree of freedom system (**Fig 1B**) was used to simulate the mechanical vibration of each heart sound component [6]. Blood masses distal to and proximal of the valve were considered as vibratory mass. Each blood mass was enclosed by viscoelastic materials including the valve and myocardial or arterial walls. These viscoelastic materials were simulated with a Voigt model [7] in which values of spring and damping factors were obtained from literature (**Table 1**) [8–11]. Springs (k) represent the elasticity and dampers (c) are responsible for the energy dissipation of material. The effective blood masses in the atria and ventricles were obtained by multiplying the real time CircAdapt-derived chamber volumes by the blood density. The effective mass of the arterial blood column was set to 0.195 kg [12].

**Table 1:** Mechanical properties of tissues.

	Spring constant (10^5^ Pa)	Damping constant (10^4^ Pa.s)
Myocardium	0.3 [9]	0.04 [9]
Aorta / pulmonary artery	1.5 [10]	2.3 [10]
Aorta / pulmonary valve	50 [11]	390 [11]
Mitral / tricuspid valve	60 [8]	2 [8]

A force (f(t)) corresponding to the real-time pressure difference across the valve is applied to the vibratory mass at the time of backward flow towards the valve. Eq (1) shows the system’s equation of motion derived from Newton’s second law [6]:

$$\left[M]\ddot{\vec{x}}(t)+[C]\dot{\vec{x}}(t)+[K]\vec{x}(t)=\vec{f}(t\right)$$ (1)

$$[M]=\left[\begin{array}{cc} m_{prox} & 0\\ 0 & m_{dis} \end{array}\right]$$

$$[C]=\left[\begin{array}{cc} c_{prox}+c_{v} & -c_{v}\\ -c_{v} & c_{v}+c_{dis} \end{array}\right]$$

$$[K]=\left[\begin{array}{cc} k_{prox}+k_{v} & -k_{v}\\ -k_{v} & k_{v}+k_{dis} \end{array}\right]$$

$$\vec{f}(t)=\left\{\begin{array}{c} 0\\ f(t) \end{array}\right\}$$

$$\vec{x}(t)=\left\{\begin{array}{c} {x(t)}_{prox}\\ {x(t)}_{dis} \end{array}\right\}$$

where [M] is the blood mass matrix, [C] the damping factor matrix and [K] the elasticity factor matrix. $\vec{x}(t)$ is the displacement vector of the blood masses and $\vec{f}(t)$ the driving force.

Because the analytical solution of Eq (1) for the transient and non-harmonic force is difficult to obtain due to coupling of variables *x*(*t*)_*prox*_ and *x*(*t*)_*dis*_, we used a numerical approach that involved four constants of integration. In order to have a pure vibration excited by the driving force, we assumed the initial velocity and displacement of the masses ${(x}_{i}(t=0)\;and\;{\dot{x}}_{i}(t=0),respectively)$ to be zero. This implies that exactly after valve closure, a valve is at equilibrium state and nothing except the driving force will drive vibration.

A numerical approach with central difference method was applied to solve the final equation below (see **S1 Text** for a complete description of the applied method):

$${\vec{x}}_{i+1}(t)={\left[\frac{1}{{\left(\Delta t\right)}^{2}}[M]+\frac{1}{2\;\Delta t}[C]\right]}^{-1}[{\vec{F}}_{i}-(\left[K]-\frac{2}{{\left(\Delta t\right)}^{2}}[M\right]){\vec{x}}_{i}-(\frac{1}{{\left(\Delta t\right)}^{2}}[M]+\frac{1}{2\;\Delta t}[C]){\vec{x}}_{i-1}]$$

$${\ddot{\vec{x}}}_{0}={\left[M\right]}^{-1}({\vec{F}}_{0}-[C]{\dot{\vec{x}}}_{0}-[K]{\vec{x}}_{0})$$ (2)

This equation was solved during valve closure in CircAdapt. Estimated displacements were considered to be vibrations representative of heart sounds. Note that all hemodynamic factors needed, including pressure and volume, were obtained real-time from CircAdapt.

### Simulation of cardio-thoracic acoustic system

A cardio-thoracic acoustic system model was used to impose the filtering effects of thoracic tissues on simulated heart sounds. Based on literature [13] we designed low-pass and high-pass digital filters in MATLAB for the M1 and T1 components of S1 and for the A2 and P2 components of S2, respectively. These filters attenuate M1 and T1 within 20 and 100 Hz by 30dB and above 100 Hz with a slope of -12dB per octave. The filters for A2 and P2 attenuate the sound within 20 and 100 Hz by 46dB and above 100 Hz with a slope of -6dB per octave. These filters were used to enable comparison between simulated heart sounds and recordings on the chest.

### Simulation of rest and exercise

To examine model functionality in various conditions and to define some features of heart sounds as indicators of cardiac condition, we simulated cardiovascular mechanics and hemodynamics-driven heart sounds in rest (control) and during exercise for the normal heart as well as for hearts with biventricular, left ventricular or right ventricular heart failure (BiVHF, LVHF and RVHF, respectively). The diameter and wall mass of cardiac chambers and large blood vessels were adapted to the level of exercise as defined previously [14]. Exercise was simulated by increasing heart rate (HR) and cardiac output (CO) from rest (4 L/min) to intense exercise (18 L/min) with steps in CO of 2 L/min. The SV (stroke volume)-CO relationship is based on measurements from healthy non-trained adults [14]. A level of moderate exercise is defined as CO = 15 L/min, HR = 141 bpm, whereas any CO > 15 L/min is regarded as intense exercise. Homeostatic pressure-flow regulation was achieved by modulation of both the systemic peripheral vascular resistance and circulating blood volume to maintain mean arterial pressure at 91 mmHg and CO at each level of exercise intensity during the adaptation process.

### Simulation of heart failure

LV and RV myocardial active stress were reduced from normal condition 100% to 50% at steps of 10% resulting in five heart failure conditions. For BiVHF this was done for all walls, for LVHF for the left ventricular free wall and septum, and for the RVHF for the right ventricular free wall.

### Animal experiments

Heart sounds were obtained from open-chest sacrifice experiments of five pigs (weight: 64 ± 1 kg) Following atrioventricular block induction by radiofrequency ablation, atrio-biventricular pacing was imposed with a fixed atrial to ventricular pacing delay (150 ms). The data-acquisition system consisted of a custom made triaxial accelerometer located on the anterior RV base to obtain heart sounds, ECG leads as well as pressure- and volume catheters. Data were recorded on custom made data acquisition systems (IDEE Maastricht University / Maastricht Instruments BV). Data were sampled with 1000 Hz. A second-order Butterworth bandpass filter of 40–250 Hz was applied to the accelerometer signal. The displacement obtained by integration of the filtered accelerometer signal was used to compare with our CircAdapt-simulated heart sounds. S1 and S2 localizations in the displacement signal were identified with reference to ECG lead II.

### Phonocardiogram data

Simulated heart sounds were compared with phonocardiograms (PCG) of normal human subjects, recorded on the chest and available in PhysioNet dataset, an open access heart sound database, collected for an international competition [15]. The accurate positions of major heart sound components (S1 and S2) were identified using hidden semi-Markov model (HSMM) [16]. The algorithm is a statistical framework which computes the probability of being in any hidden state (S1, systole, S2, diastole) by features obtained from the heart sound. We used the same algorithm proposed in [17] to separate S1 and S2 automatically. First, the recordings were resampled up and down to 1000 Hz, then they were band-pass filtered between 25 Hz and 400 Hz and finally any spikes were removed. The frequency spectra of S1, S2 and HS were estimated using a Hamming window and the discrete-time Fourier transform. In addition, major frequency range of S1 and S2 was measured by the mean of cycles’ maximum power frequency for each recording.

## Results

### Normal heart sounds

**Fig 2:**
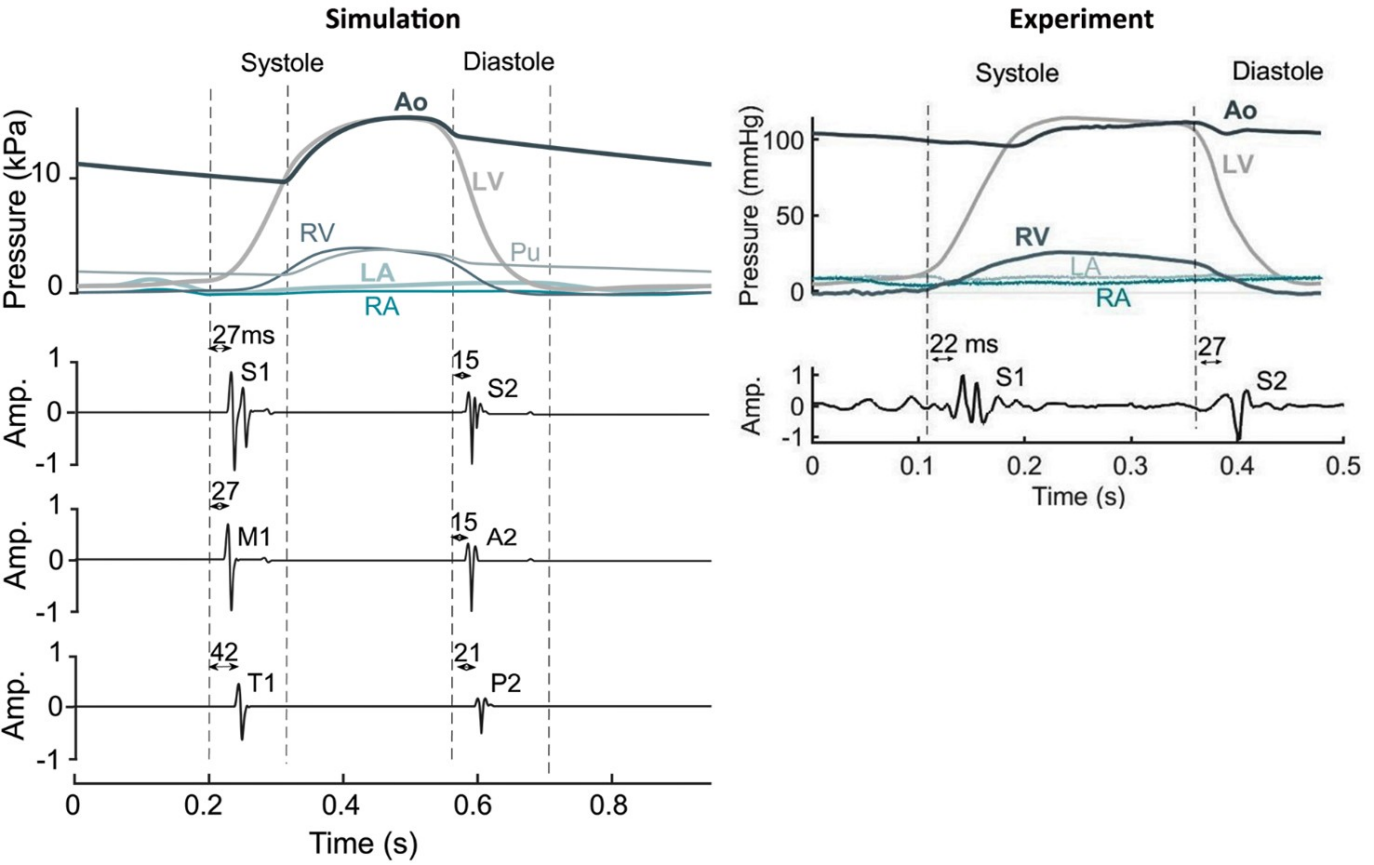
Heart sounds and their relationship with cardiovascular hemodynamics under resting conditions in simulation and recording on myocardium. Dashed lines indicate beginning/ending of LV isovolumic contraction/relaxation phase. In experiment, volumetric data was used to estimate start of isovolumic phases. Since the sensor was remotely placed from the heart which caused a delay in the Ao pressure, the Ao-LV pressure cross-over could not be used as an indicator of aortic valve closure. **Ao**: Aorta, **LA**: Left atrium, **LV**: Left ventricle, **Pu**: Pulmonary artery, **Ra**: Right atrium, **RV**: Right ventricle, **S1**: first component of heart sound, **S2**: second component of heart sound, **HS**: heart sound, **M1**: mitral component of first heart sound, **T1**: tricuspid component of first heart sound, **A2**: aortic component of second heart sound, **P2**: pulmonary component of second heart sound.

Heart sounds and their relationship with cardiovascular hemodynamics in resting condition are presented in **Fig 2**. The left panel shows simulation results. Heart sound components generated in the left side of the heart (M1, A2) preceded the ones (T1, P2) from the right side (15ms delay between M1 and T1 and 6ms between A2 and P2). Left sided components also had higher intensity than the components generated in the right heart. The delay in the simulated heart sounds with respect to pressure crossover and valve closure was in line with previous reports on clinical data as well as with our animal experimental data presented in the right panel [18–23]. S1 occurred with a 27 ms (simulation) and 22 ms (experiment) delay after mitral valve closure. Also, S2 occurred 15 ms (simulation) and 27 ms (experiment) after aortic valve closure. The experimentally measured heart sounds showed similar morphology to the simulated heart sounds. Audio files of the simulated heart sounds can be obtained in S1 Audio.

**Fig 3:**
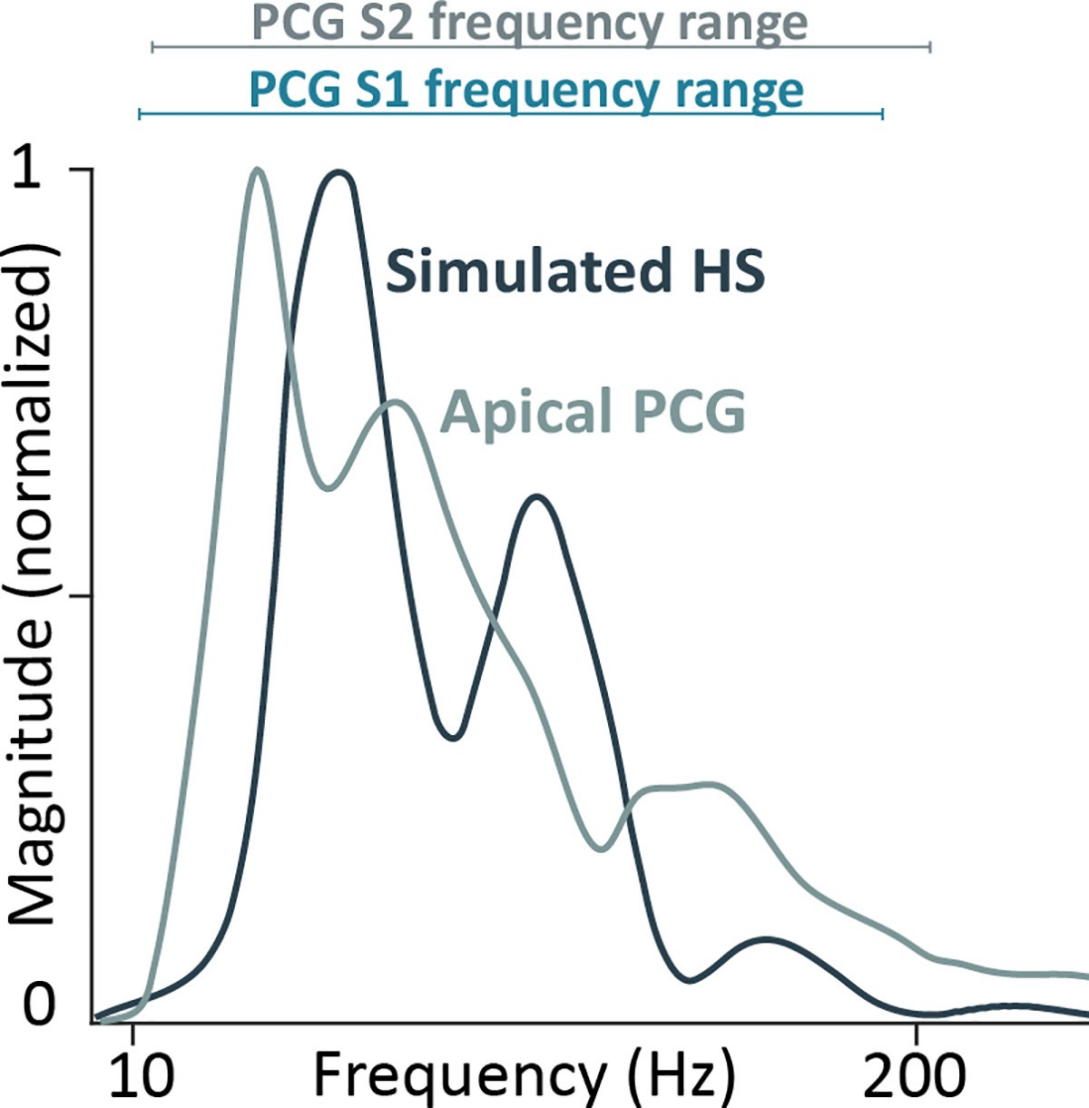
Frequency spectrum of simulated normal heart sound and its comparison with the pattern of PCG recorded on apical area and with frequency ranges of S1 and S2.

**Fig 3** shows the frequency spectrum of the simulated heart sound after transmission through the thorax in comparison with the recordings of the PhysioNet dataset. Simulated heart sounds and the mean of apical phonocardiographic recordings showed similar magnitude-frequency distributions with dominant frequencies roughly between 10 and 200 Hz.

### Heart sounds during heart failure

**Fig 4:**
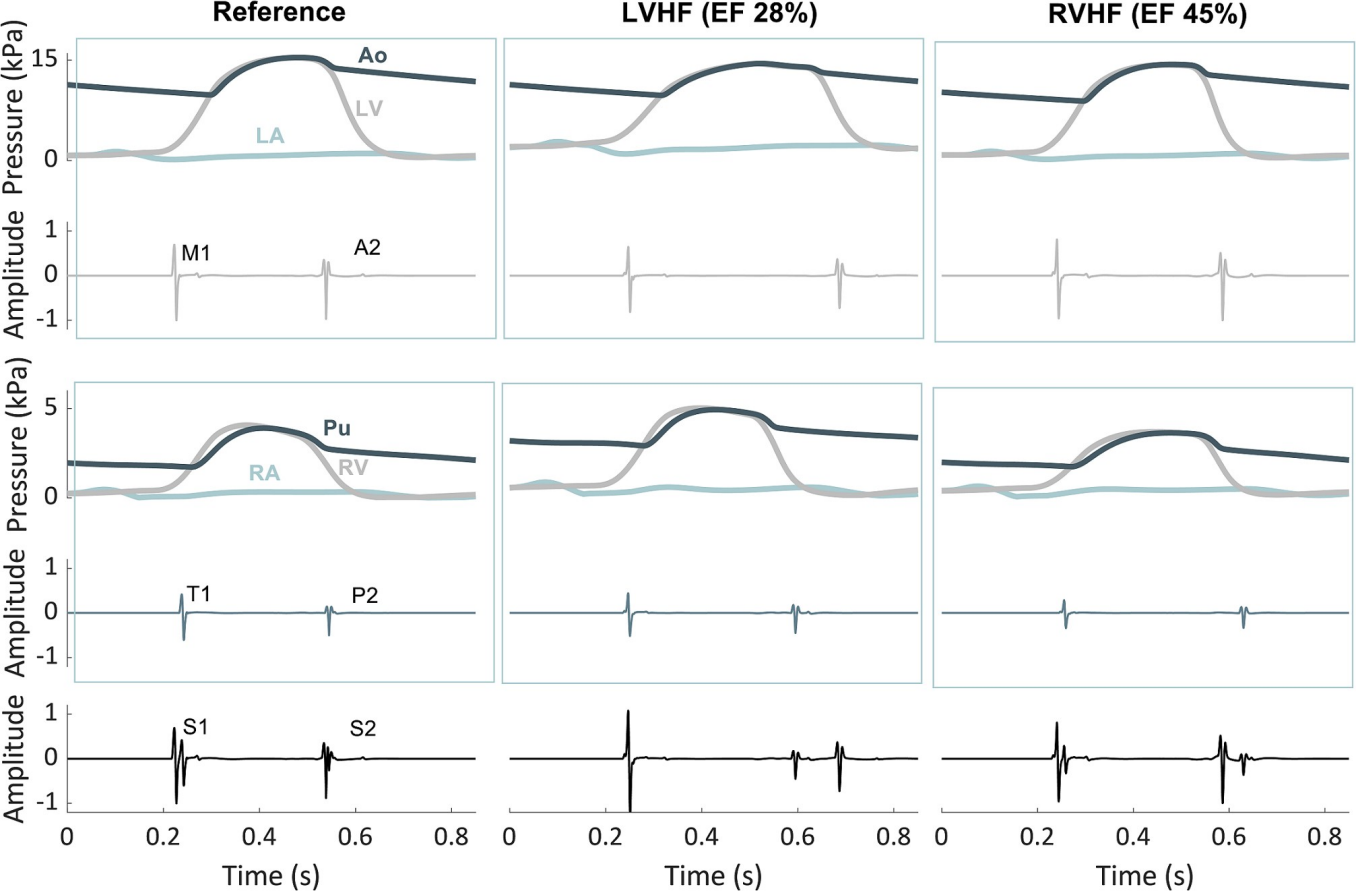
Simulated heart sounds and pressures at reference and left/right heart failures. **EF**: ejection fraction.

Simulated heart sounds and corresponding pressures for normal condition (reference) as well as severe left (LVHF) or right (RVHF) ventricular heart failure (LV ejection fraction of 55%, 28% and 45%, respectively) are presented in **Fig 4.** In LVHF, the depressed contractility of the LV caused a significant decrease in magnitude and rate of rise of left ventricular pressure, resulting in lower amplitude of M1. A2 had a lower amplitude because of not only lower Ao-LV pressure difference at the time of backward flow towards the valve but also because of higher end systolic LV volume. Enhanced blood mass and lower driving force together generated smaller A2 amplitude. Similar amplitude reduction was noticeable for T1 and P2 in RVHF. Additionally, simulated LVHF was accompanied with increased end diastolic LV volume and, hence, with a prolonged LV contraction in LVHF, causing a delayed A2 and consequently fusion and even reverse splitting of S2 [24].

### Heart sounds during exercise

**Fig 5:**
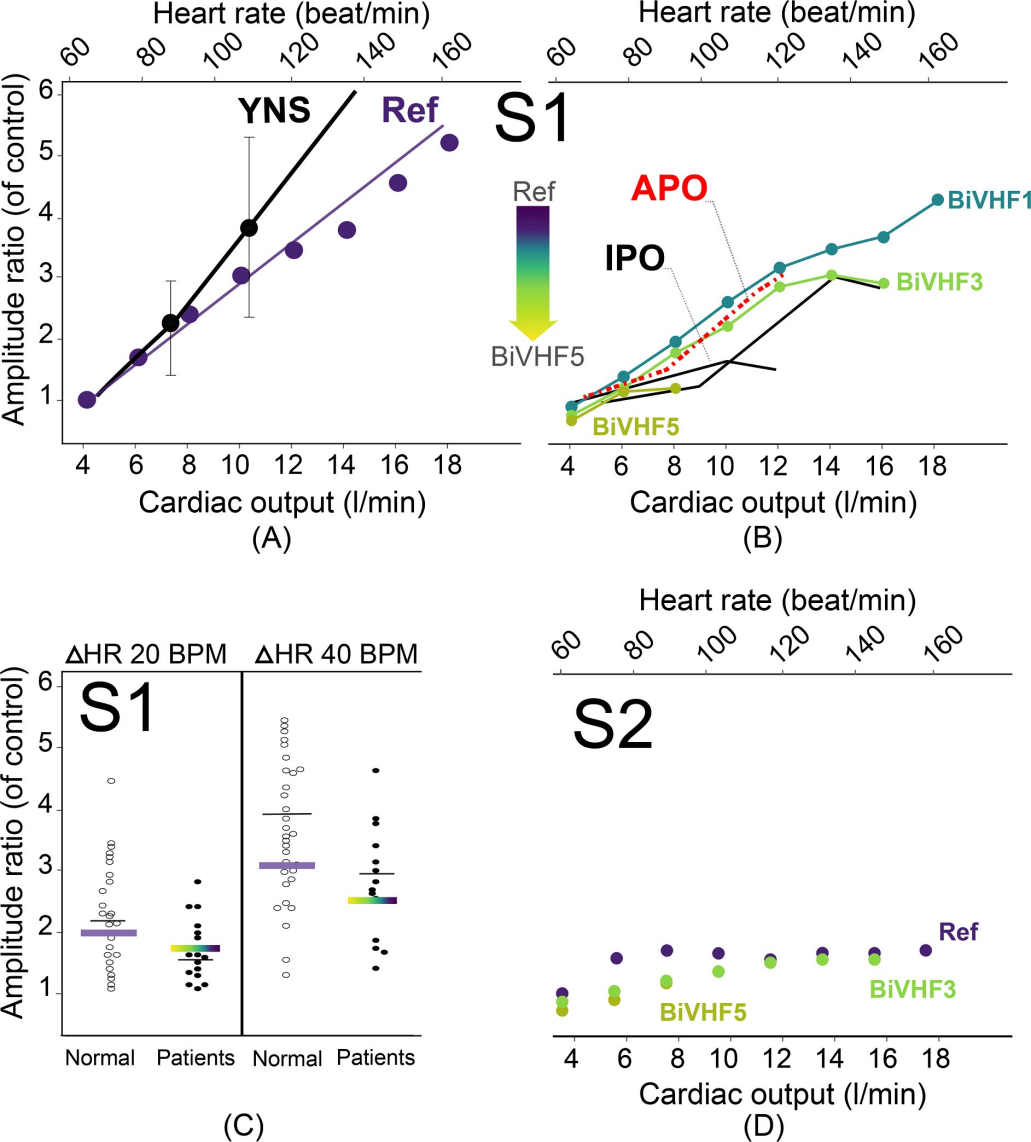
Relationship between heart rate and amplitudes of S1 and S2. The amplitudes are normalized to the control. All the experimental data (black dots and lines) are adapted from the figures in the article by Bergman and Blomqvist 1975 [25] in which patients have at least one major coronary artery with reduced vessel diameter by 50%. (a) A linear relationship between S1 amplitude and exercise level in normal condition for both simulation and experiment. (b) Simulated biventricular heart failure causes a reduction in S1 amplitude which is in agreement with the abnormal experimental data. (c) Average of S1 amplitude for both normal and abnormal simulations is within the range of experimental data. (d) Exercise doesn’t change S2 amplitude significantly compared to S1. **APO**: average of patient observations, **BPM**: beat per minute, **BiVHF1-5**: biventricular heart failure levels, **HR**: heart rate, **IPO**: individual patient observations, **Ref**: reference, **YNS**: young normal subjects.

**Fig 5A** demonstrates the relationship between the amplitude of the first heart sound and heart rate / cardiac output. Results of the simulations for the normal heart were in line with the observation on young healthy subjects in a clinical study by Bergman et al. [25]. With increased cardiac output or exercise level, the maximum rate of rise of LV pressure (dp/dt_max_) increased and, consequently the magnitude of S1 [13]. The amplitude of S1 was positively related to exercise level (R^2^:0.99, p<0.001), whereas S2 amplitude did not change significantly with exercise. Fig 5B shows simulation results as well as clinical data for various levels of exercise in the presence of BiVHF (simulations) or coronary artery disease (patients). Though S1 amplitude in these pathologic conditions also increased with level of exercise, the slope of this relation decreased. Fig 5C shows the S1 amplitude ratio between rest and increases in heart rate of 20 and 40 bpm, respectively, for normal subjects and patients. Simulation results were close to the means of the observational data. Fig 5D shows that S2 amplitude barely changes with increased levels of exercise, both for simulations of the normal heart as well as for BiVHF simulations.

**Fig 6:**
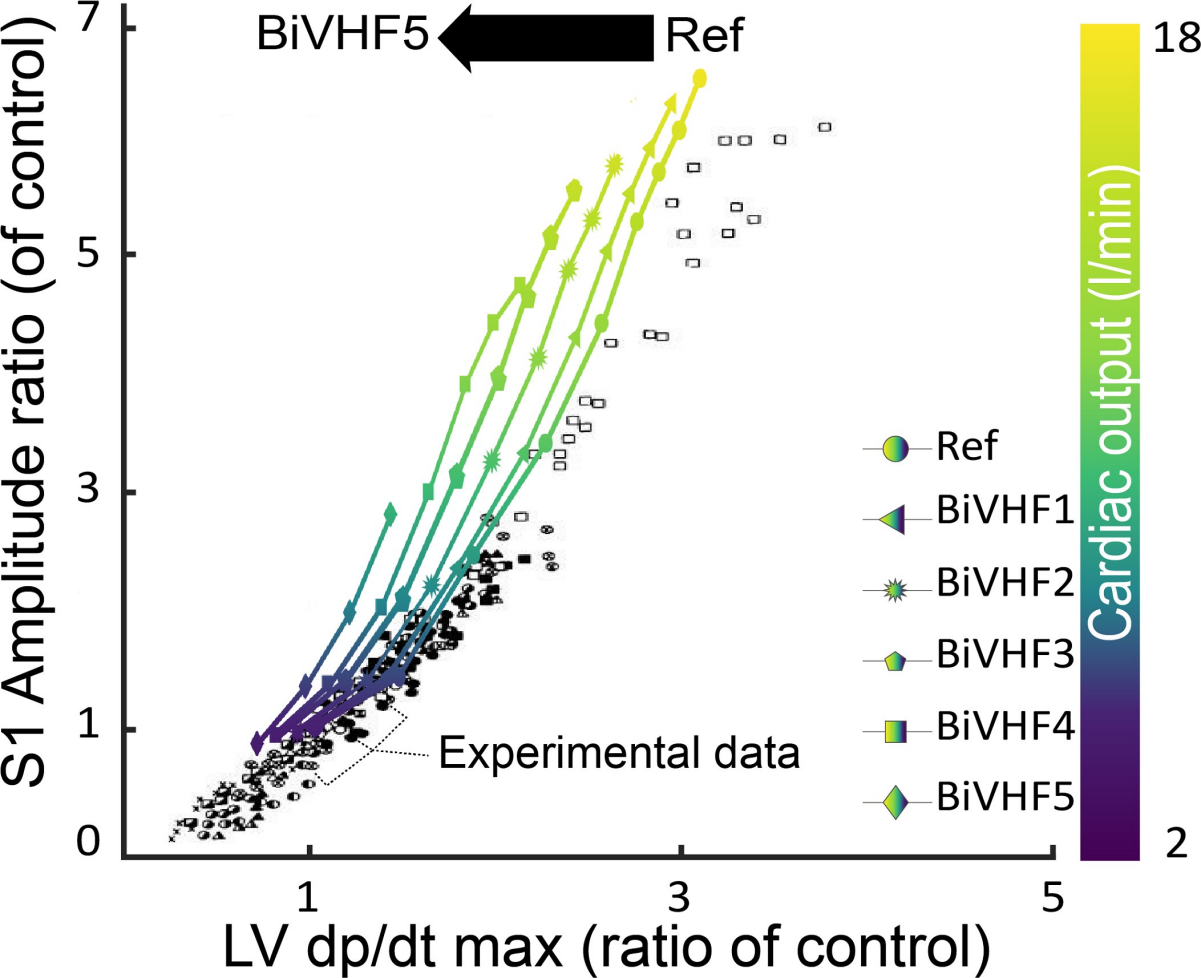
Relation between LV dp/dt_max_ and the first heart sound amplitude in simulations and reported experimental observations [26]. Conditions used in the experiment are: Aortic insufficiency, Aortic occlusion, Atropine, Artery occlusion, Epinephrine, Haemorrhage, Histamine, Isoprenaline, Methoxamine, Myocardial infarction, Norepinephrine, Phenylephrine, Pulmonary Rapid saline infusion, Pitressin, Veratridine, Venae cavae occlusion. **BiVHF1-5**: biventricular heart failure levels, **LV dp/dt max:** maximum of first derivative of left ventricular pressure, **Ref**: reference.

**Fig 6** compares the experimental data of diverse (pathological) conditions (Intravenous administration of various drugs, mechanical obstruction of great vessels, myocardial infarction, aortic insufficiency, hemorrhage, infusion of saline) by Sakamoto et al. [26] and the simulation data for different levels of BiVHF and exercise. In simulation data, a linear relationship was observed between LV dp/dt_max_ and S1 amplitude (R^2^:0.91, p<0.001). Heart failure caused a reduction in dP/dt_max_ and consequently a reduction in S1 amplitude. Hence, while the relation between S1 amplitude and LV dp/dt_max_ remained linear during heart failure, it shifted to the left. This leftward shift was proportional to the severity of simulated heart failure.

**Fig 7:**
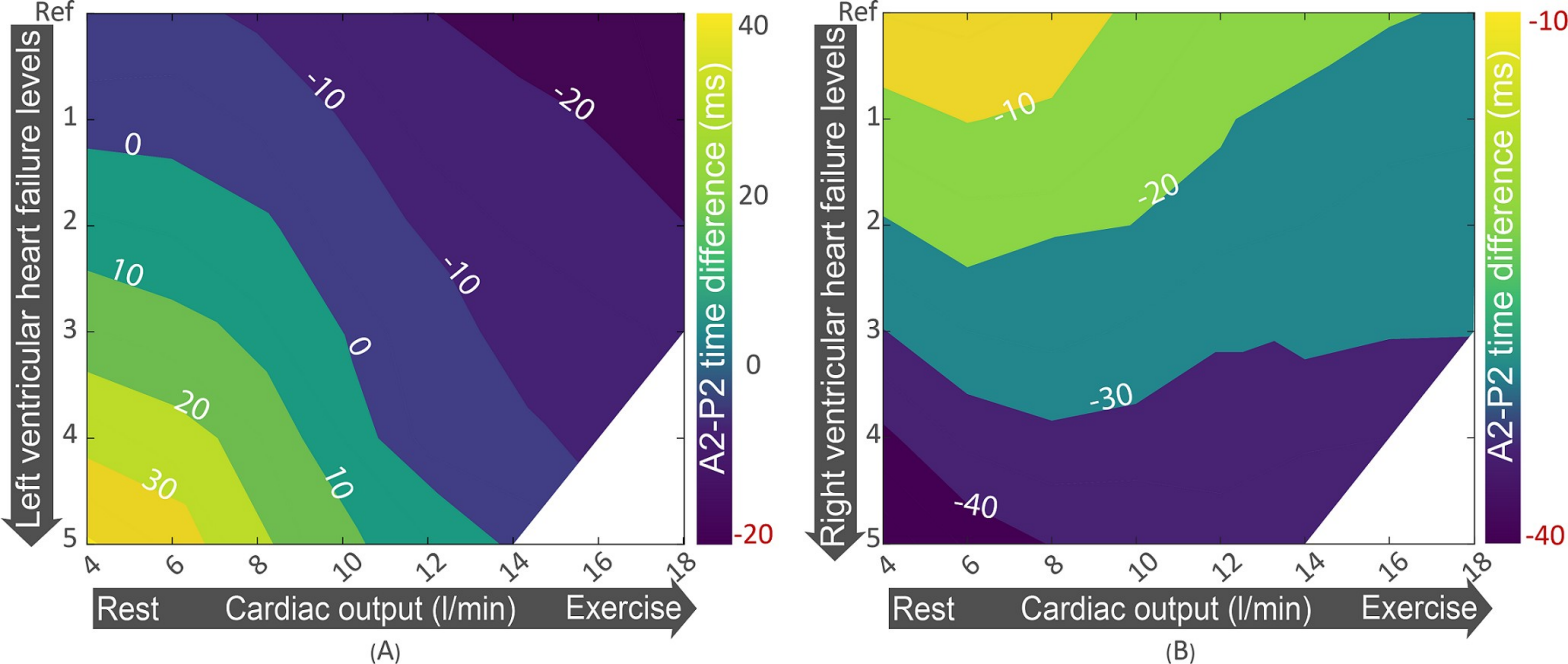
(a) A2-P2 splitting interval for left ventricular heart failure of various severity levels and exercise levels. (b) A2-P2 splitting interval for right ventricular heart failure of various severity levels and exercise levels. Positive values indicate reverse splitting.

A2-P2 splitting interval for left and right heart failure on various exercise levels is shown in **Fig 7.** Positive intervals indicate reverse splitting of S2, and negative intervals indicate normal splitting. Whereas reverse splitting in LVHF was related to both cardiac output and LVHF severity, the splitting interval did not depend on RVHF severity. Splitting was the largest in resting condition when the level of heart failure (both left and right) severity was the highest.

## Discussion

We present a novel mechanical vibration model of heart sound generation implemented in CircAdapt, a lumped-parameter real-time computational model of the cardiovascular system [5]. To the best of our knowledge, this is the first hemodynamic-based heart sound generation model embedded in a complete real-time computational model of the cardiovascular system. Simulated heart sounds are similar to experimental and clinical measurements, both quantitatively and qualitatively. Three major findings of our simulations are: i) S1 amplitude significantly increases during exercise, while the change in S2 amplitude is not significant; ii) BiVHF causes a reduced S1 amplitude compared to the normal condition with the same exercise level; and iii) splitting of S2 is narrowed/even reversed in LV heart failure, and prolonged in RV heart failure. In heart failure, the quantitative acoustic behavior of heart sounds with different cardiac output and/or exercise levels could be used to find out new biofactors for classification of different severity levels of systolic heart failure.

The model replicated not only the observed abovementioned linear relationships, but also paradoxical experimental findings, like the unchanged S2 amplitude during exercise, indicating the appropriate physics and physiology implemented in the model.

The energy content of a mechanical vibration is proportional to the amplitude squared. This energy represents hemo-mechanical energy of the heart contraction transformed into vibrations. Since lower amplitudes of S1 signify lower energy contents of the wave, we propose to use S1 amplitude as a proxy for cardiac contractility or dp/dt_max_. Although right-sided generated heart sound components contribute less to S1 and S2 than their left-sided equivalents, superposition of the waves can cause constructive or destructive interference on final S1 or S2 that depends on timing of the components in various conditions.

We found that heart sounds generated from the same left ventricular stroke volume but with different contractility levels can have different S1/S2 amplitude, which is in agreement with studies on various cardiac conditions [26]. Additionally, we couldn’t find an explicit relationship between amplitudes and both diastolic and systolic ventricular and aortic pressures, which is also consistent with the literature [26]. In addition, although exercise may increase arterial pressure, this increased pressure doesn’t change S2 amplitude significantly in comparison to the increase in S1 amplitude.

The experimental data of the patients with coronary artery disease showed a smaller increase of S1 amplitude during exercise as compared to normal controls. A similar trend was seen in the simulated data for high degrees of BiVHF. Additionally, normal conditions with different cardiac outputs showed a continuous increasing trend in both experimental and simulation results [25].

### Comparison with other heart sound models

Previous studies have simulated aortic and mitral components of heart sounds. However, these studies focused on one valve and one heart sound component at a time, and the heart sound model was not integrated in a complete model of cardiovascular system capable of simulating various cardiac conditions [8,12,27–29] These studies also did not compare between results of simulation and recordings of heart sounds on the heart and chest in terms of amplitude, frequency and timing for various conditions. Moreover, it was assumed that vibration of the valve alone generated the heart sound, whereas at that time the idea of solitary valvular origin of heart sounds was already refuted [19–22].

### Possible future applications

The good agreement between model simulations and experimental data indicates the correctness of the theory and assumptions, on which the model is based. We assumed that not only valve closure but also the surrounding structures are involved in heart sound generation. While our system is simple, it is realistic for real-time simulation of cardiovascular hemodynamics and mechanics, including heart sounds, and can be executed with different conditions. Our model provides the flexibility to manipulate hemodynamic and anatomical variables in order to obtain the resulting heart sounds corresponding to different cardiovascular conditions and to assess the effective pathophysiological factors. Our model can be used to investigate the relationships between acoustic features (frequencies, amplitudes and timings) of heart sounds on the one hand and hemodynamic factors (blood pressure, flow, velocity), anatomical parameters (inertance of the column of blood, cross-sectional areas of the valves, stiffness and elasticity of the materials) on the other hand. Because our model enables to assess the four heart sound components (M1, T1, A2 and P2) separately before they are contaminated with noise from different sources, better understanding about amplitude and timing of each component and their relationship with the corresponding hemodynamic factors can be obtained. This is almost impossible in real recordings because recorded heart sound components are vulnerable to sensor types/recording techniques and are already affected by acoustic interactions of cardiac structure in terms of interference, attenuation, reflection, and absorption. Finally, our model can be used for teaching auscultation.

### Limitations

Similar to other reported heart sound models [8,12,27–29], some assumptions were made to simplify the modeling in order to achieve fast simulation. CircAdapt is essentially a lumped parameter model. Therefore, we used a one-dimensional-two-degree-of-freedom system to simulate heart sounds, so lacking three-dimensional analysis. Also, the effect of the anatomy was based on generic properties. Obviously, heart sounds are unique for each person in terms of morphology and frequency due to different mechanical properties of cardiac structure, hemodynamic factors, BMI, age etc. [30]. Hence, an exact quantitative comparison between experimental data and simulations is not achievable. However, in the future, it can be imagined that patient-specific anatomical data can be added to achieve a more patient-specific approach. Moreover, viscoelastic behavior of biological tissues is non-linear and usually dependent on strain rate and frequency, but in this paper, they were assumed to be constant during heart beats. Finally, since the third/fourth heart sounds and murmurs have different generation mechanisms, the proposed model cannot be used for them.

## Conclusion

In conclusion, our hemodynamics-driven mathematical model provides fast and realistic simulations of heart sounds under various conditions and may be helpful to find new indicators for diagnosis and prognosis of cardiac diseases.
